# Une enquête socio-anthropologique à l'appui de la communication sur le Covid-19 En Afrique de l'Ouest

**DOI:** 10.48327/MTSIMAGAZINE.N1.2021.106

**Published:** 2021-07-26

**Authors:** B. Seytre, C. Barros, P. Bona, K. Blahima, A. Rodrigues, O. Varela, B.M. Yoro, B. Fall

**Affiliations:** 1 bnscommunication, 7 rue Ledion, 75014 Paris, France; 2 Université du Cap Vert, Cap Vert; 3 Association for Sustainable Development, Freetown, Sierra Leone; 4 Institut des Sciences des Sociétés, Centre Muraz, Bobo-Dioulasso, Burkina Faso; 5 Bandim Health Project, Bissau, Guinée-Bissau; 6 Université du Cap Vert, Cap Vert; 7 Université Houphouët-Boigny, Abidjan, Côte d'Ivoire; 8 Organisation Ouest Africaine de la Santé, Abuja, Nigeria

**Keywords:** Covid-19, Communication, Enquête socio-anthropologique, Afrique de l'Ouest, Covid-19, Communication, Socio-anthropological survey, West Africa

## Abstract

L'adhésion de la population aux mesures de prévention est cruciale pour le succès de la lutte contre l’épidémie de Covid-19, qu'il s'agisse du respect des gestes barrières ou de la vaccination. Nous avons mené dans cinq pays de la Communauté économique des États d'Afrique de l'Ouest (Burkina Faso, Cap Vert, Côte d'Ivoire, Guinée Bissau, Sierra Leone) une enquête socio-anthropologique sur les représentations du Covid-19 susceptibles d'influencer cette adhésion. Nos résultats montrent que les messages de popularisation des gestes barrières ont extrêmement bien pénétré la population et qu'ils sont parfaitement compris, mais qu'une partie substantielle de la population nie la présence du Covid-19 dans son pays, que la transmission du SARS-CoV2 par des personnes asymptomatiques est majoritairement ignorée, que les facteurs de risque de formes graves de la maladie sont insuffisamment connus et qu'une majorité des enquêtés craint de contracter le Covid-19 en se rendant dans un centre de santé. L’« infodémie » qui circule sur les réseaux sociaux ne semble pas contribuer aux différentes idées fausses que nous avons mises en évidence, idées qui sont le produit des observations et de l'interprétation des personnes interviewées. Nous proposons une réorientation de la communication sur le Covid-19 sur la base des résultats de notre enquête.

## Abstract (en)

The population's adherence to preventive measures is crucial for the success of the fight against the Covid-19 epidemic, whether it is a question of respecting barrier gestures or vaccination. We conducted a socio-anthropological survey in five countries of the Economic Community of West African States (Burkina Faso, Cape Verde, Côte d'Ivoire, Guinea Bissau, and Sierra Leone) on the representations of Covid-19 that may influence this adherence. Our results showed that raising the awareness of barrier gestures was extremely well-received by the population and is perfectly understood, but that a substantial part of the population denies the presence of Covid-19 in their country, that the transmission of SARS-CoV2 by asymptomatic persons is mostly ignored, that the risk factors of severe forms of the disease are insufficiently known and that a majority of the respondents fear contracting Covid-19 when visiting a health center. The “infodemia” circulating on social networks does not seem to contribute to the various misconceptions we have highlighted, which are the product of the interviewees’ observations and interpretation. We propose a reorientation of communication about Covid-19 based on the results of our survey.

## Introduction

L'adhésion de la population aux gestes barrières (distanciation, port du masque, lavage des mains, pas de serrage de mains ni d'accolade) est l'un des principaux volets de contrôle de l’épidémie de Covid-19. Bien que des campagnes massives de promotion de ces mesures aient été menées et continuent de l’être dans les pays d'Afrique de l'Ouest, des articles et des études non publiées montrent qu'une fraction significative de la population ne respecte pas les principaux gestes barrières (lavage des mains, port du masque, distanciation), ce dont la presse se fait également l’écho. L'adhésion à l'ensemble des gestes barrières a été estimée à 12,3% en Éthiopie et au port du masque à 47% en Côte d'Ivoire, 42% au Burkina Faso, 35% au Niger, 32% au Nigéria et 30% au Sénégal.^1^, ^2^, ^3^, ^4^

L'Organisation Ouest Africaine de la Santé (OOAS), agence de la Communauté économique des États d'Afrique de l'Ouest (CEDEAO), nous a demandé de mener une étude socio-anthropologique dans cinq de ses États membres et de proposer, si besoin, des adaptations des messages de communication sur la base des résultats de cette enquête. L'objectif de l’étude était ainsi, spécifiquement, de fournir une image de la culture en santé de la population sur le Covid-19, « culture en santé » étant la traduction que nous proposons du concept de « *health literacy* »^5^.

La communication doit-elle s'attacher à améliorer cette culture en santé et, si oui, sur quels aspects? L’« infodémie », néologisme datant d'une vingtaine d'années qui compare la diffusion de fausses informations à une épidémie parallèle, aurait-elle entraîné une désinformation sur les gestes barrières, promu des idées fausses qui entraveraient l'adhésion aux messages de santé publique ?^6^ La communication doit-elle apporter des réponses et, si oui, lesquelles? Telles étaient les questions auxquelles l'enquête devait répondre.

Le succès de la vaccination contre le Covid-19, qui a commencé en février 2021 en Afrique de l'Ouest, repose notamment sur l'acceptation de cette vaccination par la population, ce qui double l'enjeu de la compréhension des raisons du non-respect des gestes barrières, car nous pouvons faire l'hypothèse que ces raisons contribueront également à un éventuel désintérêt envers la vaccination.

## Méthodes

En accord avec l'OOAS, nous avons sélectionné cinq pays de la CEDEAO choisis pour leur diversité de langue, de taille, de développement économique et d'environnement géographique: Burkina Faso, Cap Vert, Côte d'Ivoire, Guinée-Bissau et Sierra Leone, ce pays ayant, en outre, connu une épidémie de maladie à virus Ébola en 2014-2016. Nous avons mené du 16 octobre au 20 novembre 2020 une enquête quantitative en face-à-face auprès de 400 personnes dans des grandes villes de chaque pays, soit 2 000 au total, avec des quotas par sexe et tranches d’âges pour obtenir des échantillons représentatifs de la démographie (Tableau I)^7^.

**Tableau I:** Répartition des enquêtés par âge et sexe, informations sur leur niveau d’étude et leur activité Distribution of respondents by age and gender, information on their level of education and their activity

	Burkina Faso	Cap Vert	Côte d'Ivoire	Guinée-Bissau	Sierra Leone	Total
**Femmes**	206	199	192	215	195	1007
18-24	140	37	85	53	32	347
25-59	55	127	101	140	139	562
60 et +	11	35	6	22	24	98
**Hommes**	194	201	208	185	205	993
18-24	131	41	83	39	42	336
25-59	50	134	109	130	135	558
60 et +	13	26	16	16	28	99
**Niveau d’étude**						
Aucun	69	28	72	54	67	290
Primaire	85	73	49	57	48	312
Secondaire	204	230	119	198	168	919
Supérieur	42	69	160	91	117	479
**Emploi**						
Secteur privé	22	72	68	46	39	247
Secteur public	11	83	31	47	38	210
Secteur informel	155	72	147	144	111	629
Aucun	38	84	50	72	85	329
Retraité	3	34	9	7	22	75
Étudiant	171	55	95	84	105	510
**Total général**	**400**	**400**	**400**	**400**	**400**	**2000**

Chaque enquêteur choisissait un interviewé dans une maison ou une concession, puis en laissait cinq de côté avant de chercher un autre interviewé dans la sixième habitation. Si plusieurs personnes répondaient au critère dans un même lieu, celle qui était interrogée était choisie au hasard. Les critères d'exclusion étaient: maladie mentale, sénilité et toute maladie empêchant de répondre aux questions. Les réponses étaient anonymes. Tous les répondants étant majeurs et aucune question ne portant sur leur santé, nous n'avons pas soumis le protocole à un comité d’éthique.

Pour limiter le risque de confusion entre infection asymptomatique et symptomatique, et entre SARS-CoV2 et Covid-19, les questions à propos de la maladie mentionnaient « la maladie Covid-19 » et celles sur le virus, « le coronavirus ».

Les réponses étaient, selon les conditions du terrain, soient notées par les enquêteurs sur des formulaires imprimés puis saisies sur ordinateur dans Microsoft Forms, soient directement saisies par les enquêteurs sur des tablettes connectées. Les résultats ont été générés et traités sur Excel.

196 personnes ont refusé de répondre, dont 152 en Sierra Leone. Les motifs de non-réponse n’étaient pas toujours connus et n'ont pas été systématiquement relevés. Cependant, en Sierra Leone, lorsqu'une motivation était formulée c’était que l'enquête visait à gagner de l'argent grâce à des réponses bénévoles ou que le gouvernement voulait des informations pour demander de l'aide internationale.

Parallèlement, nous avons observé et documenté photographiquement le port du masque dans des espaces publics.

Au Burkina Faso, en Côte d'Ivoire et en Sierra Leone, nous avons conduit en janvier 2021 des entretiens qualitatifs auprès de 24 personnes identifiées lors de l'enquête quantitative comme ayant des idées erronées sur le Covid-19 et qui avaient accepté de fournir leur prénom et leur numéro de téléphone (7 au Burkina Faso, 8 en Côte d'Ivoire et 9 en Sierra Leone).

## Résultats

Nous n'indiquons généralement pas les non-réponses, ce qui explique que les totaux n'atteignent pas 100%.

### Réalité du Covid-19

Un nombre limité de personnes interviewées ont répondu non à la question « Pensez-vous que le Covid-19 est une maladie qui existe vraiment? », avec des différences notables entre pays: 2,7% au Cap Vert, 9,2% en Guinée-Bissau, 10,7% au Burkina Faso, 18% en Côte d'Ivoire et 20,2% en Sierra Leone. Cette réponse diminuait avec l’élévation du niveau d'instruction: 19,7% chez les enquêtés n'ayant pas fait d’études, et 15,4%, 9,6% et 10,6% chez les personnes ayant un niveau d’études, respectivement, primaire, secondaire ou supérieur.

Les entretiens individuels ont affiné ce que représente la croyance dans la réalité du Covid-19. Les 24 personnes interviewées estimaient toutes que la maladie est une réalité, aucune ne reprenant les théories complotistes selon lesquelles la pandémie serait une invention, mais 13 d'entre elles affirmaient que si le Covid-19 existe sur d'autres continents, il n'est pas présent dans leur pays.

### Cause et transmission

97,6% des interviewés avaient « entendu parler du coronavirus » et 78,7% ont répondu « oui » à la question « Savez-vous ce qu'est un virus? », les réponses affirmatives recouvrant, bien sûr, des connaissances très différentes (Tableau II).

**Tableau II:** «Savez-vous ce qu'est un virus?» “Do you know what a virus is?”

	Non	Oui
Burkina Faso	20,7%	79,2%
Cap Vert	13,2%	86,7%
Côte d'Ivoire	18,2%	81,7%
Guinée-Bissau	34,5%	65,5%
Sierra Leone	19,5%	80,5%
**Total**	**21,2%**	**78,7%**

Si près des trois quarts des interviewés, en moyenne, savaient qu'on peut trouver le coronavirus chez les personnes malades, moins d'un tiers estimaient que des personnes asymptomatiques peuvent être porteuses (10,2% à 50% selon les pays) (Tableau III). De façon cohérente, la très grande majorité des enquêtés pensaient que les malades peuvent transmettre le Covid-19 (de 85% en Sierra Leone à 95,2% au Cap Vert), mais 20,1% seulement savaient que c’était aussi le cas de personnes non malades (Tableau IV).

**Tableau III:** «Si je vous dis que la maladie est provoquée par un virus, un coronavirus, chez qui peut-on trouver ce virus?» “If I tell you that the disease is caused by a virus, a coronavirus, in whom can we find this virus?”

	Uniquement les personnes malades du Covid-19		Tout le monde, même des gens non malades	
	Non	Oui	Non	Oui
Burkina Faso	47,5%	48,0%	45,5%	50,0%
Cap Vert	5,0%	94,0%	60,2%	35,0%
Côte d'Ivoire	35,0%	56,7%	42,0%	47,2%
Guinée-Bissau	4,0%	84,7%	77,7%	10,2%
Sierra Leone	6,2%	82,7%	67,7%	14,2%
**Total**	**19,5%**	**73,2%**	**58,6%**	**31,3%**

**Tableau IV:** «Qui peut vous transmettre la maladie COVID-19? », Réponse à « Une personne qui n'est pas malade» “Who can give you Covid-19 disease? “, answer to “A person who is not sick”

	Non	Oui
Burkina Faso	57,5%	39,7%
Cap Vert	60,0%	37,0%
Côte d'Ivoire	77,0%	15,5%
Guinée-Bissau	90,0%	5,0%
Sierra Leone	84,7%	3,2%
**Total**	**73,8%**	**20,1%**

En réponse à la question « Qui peut vous transmettre la maladie Covid-19? » nous avons également proposé sept sources de transmissions erronées mais popularisées par des outils de communication officiels utilisés en Afrique subsaharienne^8^. Les œufs, les animaux sauvages et domestiques, les poissons, la viande et les moustiques sont considérés comme davantage à risque pour le Covid-19 que des individus asymptomatiques (Tableau V).

**Tableau V:** Réponses positives à la question « Qui peut vous transmettre la maladie Covid-19?» Positive responses to the question “Who can give you COVID-19 disease?”

	Personne non malade	Œufs	Animaux d’élevage	Chien ou chat	Moustiques	Poissons	Animaux sauvages	Viande crue	Personne malade
Burkina Faso	39,7%	32,2%	40,5%	42,5%	24,5%	30,5%	67,5%	60,5%	94,5%
Cap Vert	37,0%	31,2%	20,5%	19,5%	16,5%	46,5%	27,2%	49,5%	95,2%
Côte d'Ivoire	15,5%	8,7%	15,7%	24,0%	22,0%	5,7%	34,7%	21,5%	88,7%
Guinée-Bissau	5,0%	43,2%	39,7%	40,2%	57,2%	60,0%	43,0%	56,0%	91,2%
Sierra Leone	3,2%	1,0%	11,7%	3,2%	22,0%	1,5%	21,0%	7,2%	85,0%
**Total**	**20,1%**	**23,3%**	**25,6%**	**25,9%**	**28,4%**	**28,8%**	**38,7%**	**38,9%**	**90,9%**

**Figure 1:**
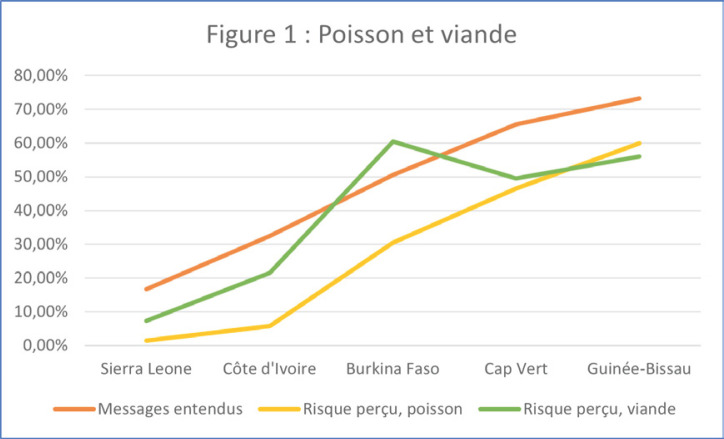
Proportion d'idées fausses corrélée à l'exposition aux messages Proportion of misconceptions correlated with message exposure

Nous avons ensuite posé la question « Avezvous entendu ou vu les messages suivants sur le Covid-19? » en citant les messages mentionnés ci-dessus. La proportion d'idées fausses est nettement corrélée à l'exposition à ces messages, dont la diffusion n'a pas eu la même intensité selon les pays (Fig. 1 et 2).

**Figure 2:**
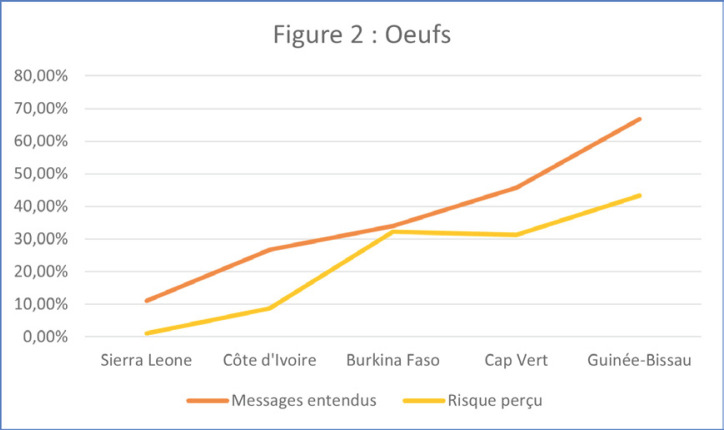
Proportion d'idées fausses corrélée à l'exposition aux messages Proportion of misconceptions correlated with message exposure

### Symptômes

La toux, les céphalées, la fièvre et les difficultés respiratoires sont des symptômes bien connus du Covid-19, avec des niveaux moyens allant de 76,5% à 86,6%, mais les symptômes spécifiques que sont la perte du goût et de l'odorat sont connus de moins des deux tiers des enquêtés, excepté au Cap Vert (Tableaux VI et VII).

**Tableau VI:** «Parmi les symptômes suivants, lesquels peuvent être dus à la maladie Covid-19?» “Which of the following symptoms may be due to COVID-19 disease?”

	Non	Oui
**Toux**	8,1%	86,6%
**Mal de tête**	16,6%	76,5%
**Fièvre**	9,5%	84,7%
**Difficultés pour respirer**	7,9%	85,9%
**Perte de l'odorat**	25,1%	61,8%
**Perte du goût**	**27,0%**	**60,5%**

Nous proposions des réponses erronées telles que « sang dans les selles », que nous ne reprenons pas ici.

**Tableau VII:** «Parmi les symptômes suivants, lesquels peuvent être dus à la maladie Covid-19?» “Which of the following symptoms may be due to COVID-19 disease?”

	Perte de l'odorat		Perte du goût	
	Non	Oui	Non	Oui
**Burkina Faso**	35,5%	57,0%	35,5%	57,2%
**Cap Vert**	5,5%	89,7%	8,2%	88,5%
**Côte d'Ivoire**	25,7%	56,5%	30,0%	53,0%
**Guinée-Bissau**	15,2%	60,5%	16,5%	60,5%
**Sierra Leone**	43,5%	45,2%	45,0%	43,5%
**Total**	**25,1%**	**61,8%**	**27,0%**	**60,5%**

### Prévention et traitement du Covid-19

Nous avons interrogé les enquêtés sur un ensemble de mesures de protection contre le Covid-19, certaines justifiées et promues par les autorités sanitaires, d'autres erronées. Pour évaluer la connaissance des gestes barrières, nous avons d'abord demandé « Avez-vous entendu ou vu les messages suivants sur le Covid-19? » puis, en cas de réponse positive, « Trouvez-vous ce conseil utile pour se protéger du Covid-19? » Quasiment toutes les personnes interrogées avaient entendu ou vu les messages, avec des niveaux allant de 93,2% à 99,7% selon les gestes barrières et les pays. Nous avons calculé les réponses positives à la seconde question, celle sur l'utilité, sur le total des personnes interviewées. Par exemple, en Côte d'Ivoire 96,2% des répondants avaient entendu ou lu un message sur la distanciation, dont 74,5% estimaient que c'est une mesure utile. Nous avons considéré que 71,7% des enquêtés jugeaient ce conseil utile (96,2% X 74,5%). Les résultats sont très élevés, avec cependant des différences selon les pays, la moyenne pour les cinq gestes barrières allant de 75,9% en Côte d'Ivoire à 98,1% au Cap Vert (Tableau VIII).

**Tableau VIII:** Pourcentage des interviewés qui trouvent le conseil utile Percentage of respondents finding the advice usefu

	Se laver souvent les mains	Garder 1 m de distance	Porter un masque	Éviter les foules	Ne pas serrer les mains	Moyenne
**Côte d'Ivoire**	81,3%	71,7%	75,7%	73,8%	76,8%	75,9%
**Sierra Leone**	81,1%	82,5%	83,6%	84,3%	83,0%	82,9%
**Burkina Faso**	93,78%	90,3%	87,1%	91,0%	91,5%	90,7%
**Guinée-Bissau**	95,8%	92,1%	95,5%	90,5%	91,6%	93,1%
**Cap Vert**	99,0%	98,0%	98,3%	98,0%	97,3%	98,1%

Nos observations de terrain montrent cependant que le masque est très peu porté, voire souvent pas du tout, au Burkina Faso, en Côte d'Ivoire, en Guinée-Bissau et en Sierra Leone dans des lieux comme les marchés et dans les transports en commun. Nous avons interrogé les enquêtés sur 14 circonstances, aliments, boissons ou traitements pouvant être vus comme susceptibles de protéger contre le Covid-19. Le climat africain était considéré comme le premier facteur de protection, avec des réponses positives allant de 39,8% à 72,3% selon les pays, suivi d'un traitement de la médecine moderne (rappelons qu'aucun traitement protecteur n'existe et que cette enquête a eu lieu avant l'annonce de la vaccination) avec 45,3% à 64,5% selon les pays (Tableau IX).

**Tableau IX:** «Les circonstances ou mesures suivantes réduisent-elles le risque d'avoir la maladie Covid-19? » (réponses « oui») “Do the following circumstances or actions reduce the risk of having COVID-19 disease?” (answers “yes”)

	Burkina Faso	Cap Vert	Côte d'Ivoire	Guinée-Bissau	Sierra Leone	Total
**Boire un désinfectant**	3,3%	1,3%	3,5%	48,3%	12,0%	**13,7%**
**Boire de l'eau de Javel**	3,5%	14,3%	1,5%	64,8%	10,0%	**18,8%**
**Boire une tisane de feuilles de neem**	28,8%	23,5%	30,8%	40,5%	12,5%	**27,2%**
**Une nourriture équilibrée**	15,8%	52,8%	16,0%	45,5%	11,5%	**28,3%**
**Être africain**	37,8%	28,5%	29,8%	31,5%	22,3%	**30,0%**
**Manger de l'ail**	16,8%	47,8%	13,3%	46,8%	33,3%	**31,6%**
**Des boissons chaudes**	23,0%	47,0%	22,5%	47,3%	18,3%	**31,6%**
**Boire du thé**	13,8%	56,8%	19,0%	59,3%	15,5%	**32,9%**
**Boire du jus de citron**	18,8%	53,5%	14,3%	55,5%	27,3%	**33,9%**
**Faire de l'exercice physique**	27,0%	41,0%	24,5%	48,5%	44,0%	**37,0%**
**Un traitement de la médecine traditionnelle**	40,0%	44,3%	41,8%	29,5%	36,8%	**38,5%**
**La prière**	47,5%	21,3%	52,5%	37,0%	71,5%	**46,0%**
**Un traitement de la médecine moderne**	49,5%	64,5%	53,0%	54,5%	45,3%	**53,4%**
**Climat chaud africain**	72,3%	39,8%	53,5%	63,3%	46,3%	**55,0%**

Les réponses sont relativement proches dans les cinq pays pour le fait d’être africain ou de pratiquer de l'exercice physique, les traitements de la médecine traditionnelle ou moderne et le climat chaud, mais sont, en revanche, très différentes pour les neuf autres choix proposés.

Ceci est particulièrement notable pour l'ingestion d'un désinfectant ou d'eau de Javel, geste qui a entraîné des cas d'intoxication souvent mortels, le taux de réponses positives allant de 1,3% à 64,8%^9^. Nous n'avons pas d'explication pour de telles différences.

### Facteurs de risque

En moyenne, 63,8% des enquêtés savent que les personnes de 60 ans et plus sont davantage à risque de Covid-19, avec des différences notables entre pays puisque c'est le cas d'un peu moins de la moitié des répondants au Cap Vert et en Sierra Leone, mais plus de 70% au Burkina Faso, en Côte d'Ivoire et en Guinée-Bissau (Tableau X).

**Tableau X:** «Est-ce que les personnes de 60 ans et + courent davantage, autant ou moins de risque que les autres d’être malades du Covid-19?» Are people aged 60 years and older at greater, equal, or lesser risk for Covid-19 disease than others?”

	Moins de risque	Autant de risque	Plus de risque
Burkina Faso	2,2%	22,5%	70,7%
Cap Vert	5,5%	46,5%	46,2%
Côte d'Ivoire	2,2%	14,2%	76,5%
Guinée-Bissau	3,5%	7,0%	78,5%
Sierra Leone	19,7%	18,7%	47,2%
**Total**	**6,6%**	**21,8%**	**63,8%**

La connaissance des risques liés à l’âge était dépendante de l’âge des interviewés. Ainsi, en moyenne sur les cinq pays, 47,2% seulement des 60 ans et plus savaient que les plus de 60 ans sont davantage à risque, alors que c’était le cas de 70,1% des 18-24 ans (Tableau XI). De façon symétrique, 56,8% des 18-24 ans savaient qu'ils sont moins à risque que les 60 ans et plus, alors que 37,1% des 60 ans et plus le pensaient (Tableau XII).

**Tableau XI:** « Est-ce que les personnes de 60 ans et + courent davantage, autant ou moins de risque que les autres d’être malades du Covid-19?» Are people aged 60 years and older at greater, equal or lesser risk than others of becoming ill with COVID-19?

	Réponses des 60 ans et +				Réponses des 18-24 ans			
	Davantage	Autant	Moins	Ne se prononce pas	Davantage	Autant	Moins	Ne se prononce pas
**Burkina Faso**	58,3%	33,3%	0,0%	8,3%	69,7%	21,8%	3,3%	5,2%
**Cabo Verde**	41,0%	52,5%	3,3%	3,3%	48,7%	43,6%	6,4%	1,3%
**Côte d'Ivoire**	72,7%	18,2%	0,0%	9,1%	74,4%	14,3%	3,6%	7,7%
**Guinée-Bissau**	52,6%	7,9%	5,3%	34,2%	85,9%	1,1%	2,2%	10,9%
Sierra Leone	34,6%	25,0%	30,8%	9,6%	64,9%	6,8%	14,9%	13,5%
**Total**	**47,2%**	**30,5%**	**10,2%**	**12,2%**	**70,1%**	**18,0%**	**4,8%**	**7,0%**

**Tableau XII:** « Est-ce que les personnes de moins de 25 ans courent davantage, autant ou moins de risque que les autres d’être malades du Covid-19?» Are people under the age of 25 at greater, equal, or lesser risk for COVID-19 disease than others?”

	Réponses des 60 ans et +				Réponses des 18-24 ans			
	Davantage	Autant	Moins	Ne se prononce pas	Davantage	Autant	Moins	Ne se prononce pas
Burkina Faso	0,0%	33,3%	58,3%	8,3%	4,4%	23,6%	66,8%	5,2%
Cabo Verde	8,2%	57,4%	31,1%	3,3%	7,7%	53,8%	37,2%	1,3%
Côte d'Ivoire	0,0%	22,7%	59,1%	18,2%	6,5%	10,1%	60,7%	22,6%
Guinée-Bissau	5,3%	21,1%	34,2%	39,5%	4,3%	21,7%	59,8%	14,1%
Sierra Leone	30,8%	32,7%	26,9%	9,6%	13,5%	39,2%	28,4%	18,9%
**Total**	**11,7%**	**37,1%**	**37,1%**	**14,2%**	**6,3%**	**25,2%**	**56,8%**	**11,7%**

Les jeunes connaissent, ainsi, très bien l'une des principales caractéristiques du Covid-19 qui est que la maladie touche préférentiellement les plus de 60 ans et rarement les moins de 25 ans, plus souvent asymptomatiques^10^.

Nous avons interrogé les répondants sur les quatre principales morbidités qui sont des facteurs de risque pour le Covid-19, le diabète, l'hypertension artérielle et les maladies cardio-vasculaires (HTA et MCV), les maladies pulmonaires et le surpoids. Dans l'ensemble des pays, le surpoids est un facteur de risque nettement moins connu que les autres, avec une moyenne de 47,2% contre 67,1%, 70,4% et 72,5% pour le diabète, HTA et MCV, et les maladies pulmonaires, respectivement (Tableau XIII). Les différences entre pays sont considérables, la moyenne pour les quatre morbidités allant de 26,2% en Sierra Leone à 81,9% au Cap Vert.

**Tableau XIII:** « Est-ce que ces conditions représentent davantage, autant ou moins de risque d’être gravement malade du Covid-19? (réponses positives à « davantage »)» “Do these conditions represent more, the same, or less risk of being severely ill with COVID-19? (positive responses to “more”)

	Diabète	HTA et MCV	Maladie pulmonaire	Surpoids	Moyenne
Burkina Faso	86,0%	85,0%	86,7%	53,5%	77,8%
Cap Vert	89,2%	90,2%	90,5%	57,7%	81,9%
Côte d'Ivoire	57,7%	67,0%	72,5%	42,2%	59,9%
Guinée-Bissau	78,7%	77,7%	81,0%	66,2%	75,9%
Sierra Leone	24,0%	32,2%	32,0%	16,5%	26,2%
**Total**	**67,1%**	**70,4%**	**72,5%**	**47,2%**	**64,3%**

### Risque de contamination perçu dans les structures sanitaires

À la question « Est-ce que je peux attraper le Covid-19 en me rendant dans un centre de santé pour une raison autre que le Covid-19? » 67,1% des enquêtés ont répondu oui, avec d'importantes différences entre pays. La plus faible défiance est en Sierra Leone, avec 41% et la plus élevée au Cap Vert avec 92,5% (Tableau XIV). Ces résultats ne reflètent probablement pas uniquement le niveau de confiance dans les bonnes pratiques des centres de santé, mais sont certainement également dépendants de la perception du risque de Covid-19 dans le pays.

**Tableau XIV:** «Est-ce que je peux attraper le Covid-19 en me rendant dans un centre de santé pour une raison autre que le Covid-19?» Can I get COVID-19 by going to a health center for a reason other than COVID-19?”

	Non	Ne se prononce pas	Oui
Burkina Faso	15,2%	2,2%	82,5%
Cap Vert	5,5%	2,0%	92,5%
Côte d'Ivoire	26,7%	11,5%	61,7%
Guinée-Bissau	33,2%	8,7%	58,0%
Sierra Leone	45,5%	13,5%	41,0%
**Total**	**25,2%**	**7,6%**	**67,1%**

## Confiance dans les sources d'information

En réponse à la question « Quelle confiance avez-vous dans ce que vous disent sur le Covid-19? », les répondants devaient appliquer un qualificatif (très méfiant, plutôt méfiant, ni méfiant ni confiant, plutôt confiant, très confiant) au gouvernement, au Président de la République, aux leaders religieux, aux parents et amis, à la télévision, à la radio et aux réseaux sociaux. Dans le Tableau XV, nous avons regroupé « très méfiant » et « plutôt méfiant », d'une part, et « plutôt confiant » et « très confiant », d'autre part, pour la moyenne des cinq pays. Les réseaux sociaux sont la source considérée comme la moins fiable dans tous les pays, avec 14,5% de confiance au Cap Vert, 29,8% en Côte d'Ivoire, 43% au Burkina Faso, 48% en Guinée-Bissau et 49,5% en Sierra Leone, tandis que la télévision est considérée comme la plus fiable, en moyenne pour les cinq pays. La confiance dans la télévision est de 42,8% en Côte d'Ivoire (1^re^ position pour les leaders religieux avec 49,5%), 55,8% au Cap Vert (1^re^ position), 58,8% en Sierra Leone (1^re^ position pour les leaders religieux avec 65,5%), 65,3% au Burkina Faso (1^re^ position), 68,8% en Guinée-Bissau (1^re^ position pour les leaders religieux avec 72,3%).

**Tableau XV:** « Quelle confiance avez-vous dans de ce que vous disent sur le Covid-19?» How confident are you in what you are told about COVID-19?”

	Méfiant	Confiant
**Télévision**	25,90%	58,25%
**Radio**	25,55%	57,20%
**Leaders religieux**	22,65%	56,55%
**Parents et amis**	23,70%	55,30%
**Gouvernement & ministère de la Santé**	30,90%	54,65%
**Président de la République**	32,75%	51,60%
**Médias sociaux**	42,70%	36,95%

La confiance dans les réseaux sociaux, où circulent à la fois des informations fiables et les rumeurs les plus fantaisistes sur le Covid-19 ne peut être assimilée à celle dans les autres médias et personnalités cités, qui tiennent sur le Covid-19 des discours relativement homogènes. Avoir confiance dans ces réseaux ne signifie pas avoir confiance dans tout ce qu'ils véhiculent. Les entretiens que nous avons menés le confirment.

Les 24 personnes que nous avons interviewées avaient été recrutées en fonction de diverses idées fausses qu'elles avaient avancées pendant l'enquête quantitative. Comme nous l'avons rapporté précédemment, 13 d'entre elles ont affirmé que le Covid-19 existe, mais n'est pas présent dans leur pays.

Interrogées sur leurs sources d'information, ces personnes qui niaient l'existence du Covid-19 dans leur pays ont toutes expliqué qu'elles fondent leur conviction, d'une part, sur ce qu'elles voient autour d'elles mais, surtout, sur la comparaison des images des chaînes de télévision du Nord et de celles de leur pays, les vidéos des premières étant souvent regardées sur internet. Elles ont toutes dit se méfier des informations qui circulent sur les réseaux sociaux:

- « Moi-même, je publie des trucs bizarres. Si moi je veux vraiment me fier à ces trucs, il faudrait dire que ce que je publie est vrai. Donc, je ne leur fais pas confiance. » (étudiant, 24 ans, Burkina Faso).
- « Au début, je croyais à l'existence du virus, mais la façon de gérer la riposte a vraiment enlevé cette confiance. » (étudiant, 22 ans, Burkina Faso)
- « Moi, j’écoute les informations qui viennent de l'extérieur, par exemple sur RFI. Au début, j’écoutais pour le Burkina la radio Oumega, on parlait de cas et on n'en a jamais vu. Même à la télé, je n'en ai jamais vu. Mais à la télé, par exemple en France, aux États-Unis, quand il y a eu des cas, des morts, ils les ont montrés. » (étudiant, 24 ans, Burkina Faso)
- « Le Covid-19 dans les autres mondes c'est réel, mais en Afrique on ne le trouve même pas. Nous on ne comprend pas. (…) Le ministère de la Santé qui nous donne des informations et les médias ivoiriens disent que ça existe. » (commerçant, 42 ans, Côte d'Ivoire)
- « En Europe, moi j'ai vu des gens dans chaque famille où ça pleure, j'ai perdu tant par le Covid, mais en Côte d'Ivoire, ici, zéro. J'ai vu à la télé, au Brésil il n'y a pas de sol pour enterrer les gens. Avez-vous déjà vu une interview où ils ont montré que dans une famille le Covid a tué des gens en Côte d'Ivoire? (…) » A propos des réseaux sociaux: « Il y a à prendre et à laisser. Tu prends ce qui est bon, celui qui est mauvais tu le jettes à ta main gauche. » (étudiant, 20 ans, Côte d'Ivoire)
- « En Europe quand on regarde France 24, toutes ces chaînes-là, elles montrent les morts, les contaminés, mais chez nous en Afrique c'est bizarre, on ne les voit jamais. (…) Google, Facebook, c'est des menteurs. » (étudiant, 21 ans, Côte d'Ivoire)
- « Je prends mes informations du ministère de la Santé, pas des réseaux sociaux (maçon, 42 ans, Sierra Leone)
- « Nous voyons à la télévision des gens qui meurent dans les autres pays. » (étudiant, 22 ans, Sierra Leone)
- « Nous n'avons pas vu de gens infectés par le corona. » (commerçante, 24 ans, Sierra Leone)
- « Quand il y avait Ébola, on voyait des exemples de gens infectés à la télévision, mais avec le corona, rien du tout. » (commerçante, 24 ans, Sierra Leone)
- « On regarde la télévision et on voit comme le corona frappe des gens en Amérique, en Chine, mais il ne touche personne dans ce pays. » (étudiante, 21 ans, Sierra Leone)
- « Je regarde les informations à la télévision et j’écoute la radio, dans le quartier. » (commerçante, 22 ans, Sierra Leone)
- « Mes sources d'information sont les réseaux sociaux et d'autres comme BBC Radio, CNN et les télévisions et médias locaux. Je m'informe aussi sur internet – Google, WhatsApp, Instagram, etc. J'ai un regard critique sur ce que je vois sur les réseaux sociaux. Je trouve difficile de savoir ce qu'on doit croire. » (commerçante, 28 ans, Sierra Leone)

Interrogées sur les motifs que le gouvernement aurait de mentir en affirmant que le Covid-19 est présent dans le pays alors que cela ne serait pas exact, quatre de ces treize personnes ont avancé des raisons financières et deux estimaient que c’était pour effrayer la population afin qu'elle respecte les gestes barrières, là encore en s'appuyant sur leur opinion des gouvernants et leurs observations, sans mentionner des informations provenant des réseaux sociaux.

## Discussion

Des enquêtes Connaissances Attitudes et Pratiques (CAP) et sondages ont été conduits dans la plupart des pays d'Afrique de l'Ouest. Cette étude est, à notre connaissance, la seule enquête socio-anthropologique sur le Covid-19 menée dans plusieurs pays africains pour analyser la pénétration des messages de communication diffusés depuis le début de la pandémie et proposer une stratégie de communication fondée sur des bases objectives.

Nos résultats montrent que de nombreuses idées fausses sont partagées sur la transmission du SARS-CoV2. Animaux domestiques et sauvages, œufs, moustiques, poisson et viande sont considérés comme des risques potentiels par environ un tiers de la population, alors que l'origine du SARS-CoV2 est un virus qui infecte des chauves-souris strictement inféodées à certaines régions asiatiques, même si la façon dont la barrière d'espèce a été franchie n'est pas éclaircie. Deux de ces idées erronées, la transmission par des moustiques et des animaux sauvages, pourraient partiellement s'expliquer par le fait que diverses maladies endémiques en Afrique soient transmises par des moustiques et des mammifères sauvages. Nous montrons, en revanche, que la croyance selon laquelle les œufs, le poisson ou la viande pourraient transmettre le Covid-19 est nettement corrélée au fait d'avoir entendu ou lu des messages sur ces prétendus risques, messages diffusés sur des canaux officiels nationaux et internationaux. La conséquence de ces idées erronées sur l'adhésion aux mesures de prévention du Covid-19 est difficile à apprécier. Nous pouvons cependant émettre l'hypothèse qu'elles relativisent l'importance des véritables gestes barrières chez les personnes qui partagent ces idées erronées et décrédibilisent la communication sur le Covid-19 aux yeux de celles qui savent qu'elles sont fausses. L’épidémie de maladie à virus Ébola en Afrique de l'Ouest, en 2014-2016 avait déjà vu prospérer ce type de communication erronée^11^.

Une partie substantielle de la population, variable selon les pays, pense que diverses plantes ou produits protègent contre le Covid-19. Si l'ail, le jus de citron, les infusions de feuilles de neem ou les boissons chaudes sont souvent considérés comme des protections contre des maladies dans des cultures africaines, il semble qu'il n'en ait jamais été ainsi pour le fait de boire de l'eau de Javel ou du désinfectant^12^, ^13^. Il est probable que les déclarations très largement diffusées du président Trump suggérant d'ingérer ces produits soient à l'origine de ces croyances. Nous n'avons pas d'explication sur les différences très importantes du niveau de ces croyances selon les pays mais pouvons noter qu'il est nettement inférieur dans les deux pays francophones de l’étude, où la population est peut-être moins influencée par l'actualité du pays anglophone que sont les États-Unis.

Nous montrons qu'une majorité de la population estime courir un risque de contracter le Covid-19 en se rendant dans un centre de santé, ce qui est susceptible de diminuer la demande de diagnostic et de prise en charge de maladies autres que le Covid-19 et d'interrompre des soins en cours.

Nos résultats mettent cependant en évidence, à notre connaissance pour la première fois, que la quasi-totalité de la population connaît les gestes barrières et, surtout, que la grande majorité d'entre elle sait que ces mesures sont efficaces pour protéger contre le Covid-19 (75,5% à 98,1% selon les pays). Le non-respect massif des gestes barrières ne se trouve donc pas du côté d'une éventuelle incompréhension de l'utilité de ces gestes. La multiplication à l'identique des messages incitant à respecter ces mesures est insuffisante, voire vouée à l’échec, et une autre stratégie de communication doit être recherchée.

Trois volets de nos résultats apportent des éléments qui contribuent à expliquer le manque d'adhésion aux mesures de prévention du Covid-19, bien que leur utilité soit comprise:

- Premièrement, nous montrons que si une grande majorité de la population pense que le Covid-19 existe, beaucoup croient, sans que nous n'ayons de données quantitatives, qu'il n'est pas présent dans leur pays. Il s'agit probablement là d'une des principales raisons du non-respect des gestes barrières car pourquoi se protéger d'une maladie à laquelle on ne se croit pas exposé?
- Deuxièmement, la très grande majorité de la population ignore que des personnes asymptomatiques transmettent la maladie, ce qui conforte un sentiment de sécurité tant qu'on ne rencontre pas de malades. L'OMS estime que 80% des infections par le SARS-CoV2 en Afrique subsaharienne sont asymptomatiques^14^.
- Troisièmement, les facteurs de risque sont insuffisamment connus, notamment le surpoids, ce qui n'incite pas les personnes à risque et leur entourage à respecter les gestes barrières. Le risque lié à l’âge est insuffisamment connu des personnes de 60 ans et plus (47,2%).

Nous montrons, enfin, que le risque lié à l’âge est bien connu des jeunes qui sont 70,1% à savoir que les personnes âgées sont davantage à risque et 56,8%, qu'ils sont eux-mêmes moins à risque. Conscients de ne pas être à risque de développer le Covid-19 mais ignorant qu'ils peuvent être infectés et transmettre le coronavirus à des personnes à risque, les jeunes doivent faire l'objet d'un plan de communication spécifique faisant appel à leur sens de la responsabilité, de la fraternité et de la famille.

L'enjeu d'une réorientation de la communication n'est pas seulement d'augmenter l'adhésion aux gestes barrières, mais aussi de garantir le succès de la vaccination car les raisons de respecter ou non les gestes barrières sont susceptibles d'influencer l'acceptation de la vaccination.

Nos résultats relativisent très fortement l'influence des informations fausses qui circulent sur les réseaux sociaux. Pas une seule des personnes avec qui nous avons eu des entretiens et qui croient que le Covid-19 n'est pas présent dans leur pays ne s'est appuyée sur des informations trouvées sur ces réseaux pour expliquer leurs positions. Elles se sont forgé cette conviction sur le fait qu'elles ne connaissent aucun malade du Covid-19 dans leur entourage et, surtout, en comparant ce qu'elles voient ou entendent sur les télévisions et les radios des pays du Nord, d'une part, et sur celles de leur pays, de l'autre. Déployer des efforts de communication pour réfuter les rumeurs qui circulent sur les réseaux sociaux apparaît donc comme inutile pour convaincre de la réalité du Covid-19, au moins dans les pays d'Afrique de l'Ouest. La priorité de la communication doit être de montrer la réalité de la maladie dans chaque pays.

## Conclusion

La connaissance et la bonne compréhension des gestes barrières sont des acquis remarquables de près d'un an de communication sur l’épidémie. Cependant, les résultats de notre étude socio-anthropologique permettent d'identifier des pointsessentiels sur les quels les connaissan ces de la population sur le Covid-19 doivent être modifiées ou améliorées. L'enjeu est crucial pour contribuer à un meilleur respect des mesures de prévention, qu'il s'agisse des gestes barrières ou de la vaccination.

Nous proposons les axes de communication suivants sur le Covid-19 en Afrique de l'Ouest:

- Montrer la réalité du Covid-19 dans chaque pays.
- Expliquer la transmission asymptomatique.
- Expliquer les morbidités à risque pour le Covid-19.
- Développer un plan de communication envers les jeunes.
- Rassurer sur la fréquentation des centres de santé.

Ces recommandations sont susceptibles de s'appliquer à l'ensemble de l'Afrique subsaharienne.

## Conflits D'intérêts

L'enquête socio-anthropologique a été réalisée à la demande de l'Organisation Ouest Africaine de la Santé et financée par le ministère français de l'Europe et des Affaires Étrangères, à travers Expertise France. Les auteurs ne déclarent aucun conflit d'intérêt.
